# Subliminal influence on preferences? A test of evaluative conditioning for brief visual conditioned stimuli using auditory unconditioned stimuli

**DOI:** 10.1098/rsos.160935

**Published:** 2017-09-20

**Authors:** Tobias Heycke, Frederik Aust, Christoph Stahl

**Affiliations:** Department of Psychology, University of Cologne, Cologne, Germany

**Keywords:** evaluative conditioning, implicit learning, cross-modal conditioning, awareness

## Abstract

In the field of evaluative conditioning (EC), two opposing theories—propositional single-process theory versus dual-process theory—are currently being discussed in the literature. The present set of experiments test a crucial prediction to adjudicate between these two theories: Dual-process theory postulates that evaluative conditioning can occur without awareness of the contingency between conditioned stimulus (CS) and unconditioned stimulus (US); in contrast, single-process propositional theory postulates that EC requires CS-US contingency awareness. In a set of three studies, we experimentally manipulate contingency awareness by presenting the CSs very briefly, thereby rendering it unlikely to be processed consciously. We address potential issues with previous studies on EC with subliminal or near-threshold CSs that limited their interpretation. Across two experiments, we consistently found an EC effect for CSs presented for 1000 ms and consistently failed to find an EC effect for briefly presented CSs. In a third pre-registered experiment, we again found evidence for an EC effect with CSs presented for 1000 ms, and we found some indication for an EC effect for CSs presented for 20 ms.

## 1. Introduction

The acquisition of preferences plays an important role in our daily life: Companies want us to prefer their products over their competitors', politicians want us to prefer them over their opponents, and governmental bodies want us to adopt a healthy lifestyle. Given the multitude of motivations to sway our preferences it is important to establish whether a person's attitudes can be shaped without her becoming aware of the procedure. Evaluative conditioning (EC) is one way in which preferences can be acquired: When an initially neutral stimulus (the conditioned stimulus or CS) is paired with a positive or negative stimulus (the unconditioned stimulus or US), it is subsequently evaluated in accordance with the valence of the US. We argue that understanding processes underlying evaluative conditioning could have a major impact on the understanding of preference acquisition in general. There are currently two dominant families of theories trying to explain the mechanisms that underlie the EC phenomenon [1]. Single-process theories propose that the acquisition of preferences can only happen in a conscious, deliberate, propositional manner [2,3]. Dual-process models, in contrast, propose that preferences can also be acquired in an automatic, non-conscious manner [4]. Whereas dual-process models posit an ‘indirect influence on propositional reasoning mediated by direct influence on associative evaluation’ [4] for which contingency awareness is not necessary, propositional single-process models posit that contingency awareness is a necessity for changes in preference to occur [3]. Whether a change in preferences can be achieved when people are unaware of the contingency between the CS and US is, therefore, a central question for theories of EC.

In support of associative processes, Olson & Fazio [5] report EC effects for participants who were unaware of the CS-US contingency. Others, however, have argued that awareness is a necessary condition for EC effects to occur: Pleyers and colleagues have demonstrated that unaware EC effects are found only for those CS-US pairs for which participants could recall the specific US that had been paired with the given CS [6]. Similarly, Stahl and colleagues [7] found that EC effects are absent when participants cannot recall the valence of the US that was paired with a given CS. An alternative explanation for these findings is that participants relied on their automatic affective response to the CS to answer questions about the paired US valence rather than reporting their actual memory [7,8]. Such an ‘affect-as-information’ account can explain the correlation of EC effects with participants' report of US valence from a dual-process perspective in which EC is in fact independent from awareness [9]. Consistent with the latter view, when using a method that avoids this problem, Hütter and colleagues found EC effects in the absence of contingency awareness [9].

Because these previous studies investigating contingency awareness as a necessary precondition for EC have relied on retrospective reports by participants, they have been the target of two methodological criticisms: First, such reports are merely correlational and thus causal inferences cannot be drawn; second, they are susceptible to forgetting. When awareness of the CS-US pair is assessed only after the learning phase [1,2], it is possible that a stimulus pair has been perceived during the learning phase but the episode cannot be retrieved during the contingency-awareness assessment at the end of the study. Awareness is therefore systematically underestimated if assessed by retrospective reports [2]. To the same effect, a recent meta-analysis also showed that awareness tests of unconscious learning effects are often underpowered [10]. The authors of this meta-analysis show that a joint analysis of awareness checks yields clear evidence for above-chance levels of awareness, a finding that contradicts the null findings reported in the investigated individual studies that were due to insufficient statistical power.

Experimental manipulations of contingency awareness may provide stronger evidence and further illuminate the discussion. One way to manipulate contingency awareness is by way of presentation duration (see Dedonder and colleagues [11] for a different approach): Briefly presented and masked stimuli may sometimes be processed without reaching consciousness [12,13]. By showing either CS or US very briefly, the complete CS-US pair cannot be perceived consciously, thereby interfering with contingency awareness. Observing EC under such conditions would support the notion that EC effects can be formed ‘independent[ly] of conscious awareness’ in associative learning [14]. A dual-process model would provide the most plausible explanation for such a finding, given that propositional theories propose that ‘associative learning is never automatic and always requires controlled processes’ [3].

Previous experiments have demonstrated EC effects with subliminal stimuli [15–19]. In those studies, however, it was the US (not the CS) that was presented subliminally. This is critical because it has been shown that the perception of valenced information may be possible even with very short presentation times ([20,21], but see [22]). If, in the above studies, the valence of the US has been processed consciously, then any EC effects in these studies can be explained by a propositional single-process model and do not require dual processes. Only very few studies have so far reported EC effects for briefly presented CSs [23], and these studies' methodologies have been criticized, for example, for employing between-subject manipulations of the CSs or of US valence [1,24]. Hofmann and colleagues conclude in their meta-analysis on EC in humans that more research is needed before any conclusions can be drawn about a subliminal EC effect [25].

A recent study in our laboratory addressed the possibility of subliminal EC in a series of experiments that attempted to overcome the above-discussed methodological problems. In this study, CS-US contingency was manipulated by varying CS presentation duration and/or masking, and awareness of brief and masked visual CSs was assessed immediately after their paired presentation with visual US images. Across six experiments no EC effects were found, despite the fact that CS identification was slightly above chance [24]. The present study aims to test whether EC effects can be found under slightly above-chance CS identification conditions, when three potential shortcomings of the study by Stahl and colleagues [24] are addressed.

### 1.1. The present study

The goal of the present set of experiments is to provide an even more stringent and fair test of EC with briefly presented CSs than Stahl and colleagues [24]. As in those studies, our goal was to improve upon the methodological shortcomings of previous studies discussed by Sweldens and colleagues [1]. In line with previous work, therefore, we manipulated positive and negative USs within participants to rule out any effects of mood induction; and we manipulated presentation conditions of the CSs rather than the USs to avoid that—even if USs are not reliably identified—conscious processing of US valence could be invoked as a single-process explanation for an EC effect.

First, note that, in the study by Stahl and colleagues [24], both the CS and the US were visual stimuli, presented concurrently and next to each other on the computer screen. This was done to increase the chances of EC effects by way of an implicit misattribution process [26], which assumes that participants first experience an affective response (elicited by the US) that is then misattributed to the CS because it happens to be the stimulus that is attended at the time the affective response occurs. Factors that supposedly increase the chances of implicit misattribution with visual stimuli are (i) relative salience of the CS, (ii) simultaneous onset of CS and US stimuli, (iii) shifts of attention (eye gaze) between CS and US, and (iv) moderately valent (‘mildly evocative’) US stimuli [26]. One problem that arises with the above method is that, when limiting presentation duration of the CS, participants' attention must be directed towards the CS during its brief presentation for it to have any chance of affecting cognition and behaviour. This may interfere with the effect of a simultaneous stimulus onset in the sense that it requires that participants process the US and CS sequentially and in that order. If that is the case, it was perhaps less likely in this procedure that participants experienced an affective response elicited by the US while they attended the CS.

On a related note, recent studies have investigated the effect of sequential versus simultaneous CS-US presentation on implicit EC effects. Results indicated that EC by way of an automatic process requires simultaneous pairing of stimuli, whereas an EC effect by propositional processes can also be found with sequential pairings [9,27]. This finding might contribute to explaining the absence of an EC effect in the experiments by Stahl and colleagues [24].

To address these problems, we used a cross-modal EC procedure in which the visually presented CS is paired with a simultaneously presented auditory US. This allows not only for a simultaneous CS-US presentation but also for a simultaneous *processing* of CS and US. Auditory USs paired with visual CSs have been used in conditioning procedures with children [28] and product preference studies [29,30]. The previously mentioned meta-analysis by Hofmann and colleagues [25] estimated that the size of a cross-modal EC effect does not differ significantly from unimodal EC effects. Using this cross-modal approach of CS-US presentation allows for a brief CS presentation and ensures that CS and US can be attended at the same time, an issue which has received only little attention in previous studies on subliminal EC.

Second, we assessed whether EC for near threshold visible CSs can be obtained not only in the presence but also in the absence of an online CS identification task. To ensure that briefly presented stimuli are not clearly visible, in two initial studies we will measure visibility during the learning phase, thereby using an online visibility criterion as in the studies by Stahl and colleagues [24]. Following every CS-US pairing, participants will be asked to identify the previously presented CS from a selection of all CSs. Using this online visibility criterion, we can assess an average identification rate for each CS for each participant. We use this estimate as a proxy for the perceptual awareness of the CS (while recognizing that this measure may be influenced by unconscious influences on familiarity as well as guessing). If we observe an EC effect for stimuli that were not identified at above-chance levels during the learning phase, this would constitute strong evidence that this EC effect was caused by associative processes and not by conscious propositional processes. If we observe no EC effect, even for CSs that were correctly identified at slightly above-chance levels, a single-process model of evaluative learning would be preferred as the more parsimonious account.

The visibility-check task has the additional benefit of directing participants' attention towards the CS, but it may also induce an analytic task set that may not be conducive for automatic EC. In addition, participants are presented with a set of several CSs as choice options in close temporal proximity with the US; this may dilute automatic EC effects because (i) affective response may be attributed to different CSs, and/or (ii) the additional CSs presented on the visibility-check task may also be associated with USs of different valence over the course of the learning phase. In sum, it may be argued that the visibility-check task may interfere with the formation of subtle CS-US associations during learning. To address this possibility, in a final study participants will work on a different task during the learning phase.

Third, in order to provide optimal conditions for obtaining even subtle EC effects, we introduced an additional, potentially more sensitive dependent measure: In a two-alternative forced choice (2-AFC) task, we are pitting two CSs against one another that were paired with USs of opposite valence. In at least one study on subliminal influence, a 2-AFC task showed a significant effect while an evaluative rating did not show an effect [31]. It seems plausible, therefore, that small and subtle EC effects might not be reflected in evaluative ratings because they were not perceived as large enough to justify selection of a different point on, say, a 7-point scale—but may nevertheless tip the scale when being forced to choose between two CSs that were paired either with a positive or a negative US.

As in Stahl and colleagues [24], we wanted to be certain that automatic processes have a fair chance to operate and to produce EC effects. In the study at hand we therefore realized near-threshold but slightly above-chance (instead of fully subliminal) presentation conditions. We can therefore be certain that the brief visual CS stimuli could in fact be processed, and that automatic processes were given a fair chance to operate on them.

Taken together, across three experiments we investigated EC effects for clearly visible as well as for near-threshold visual CSs that were paired with auditory USs. In the unregistered Experiments 1 and 2, we used an online visibility-check task as an index of contingency awareness; the pre-registered Experiment 3 tested whether near-threshold cross-modal EC can be found in the absence of an online visibility check. In Experiments 1 and 3, a 2-AFC choice measure was administered to test whether EC with briefly presented CSs can be found on this potentially more sensitive measure.

## 2. Unregistered Experiment 1

The first experiment tested if we can observe a cross-modal EC effect with CSs presented for 1000 ms as well as with CSs presented for 17 ms, while strictly assessing CS visibility during the learning phase.

### 2.1. Methods

#### 2.1.1. Design

In Experiment 1 we manipulated the presentation time of the CS (17 ms versus 1000 ms) as well as US valence (positive versus negative) within participants. We manipulated the order of dependent measures (2-AFC or rating first) between participants.

#### 2.1.2. Sample

An *a priori* power analysis for a paired one-sided *t*-test with *α* = *β* = 0.05 and *d* = 0.3, which is approximately the effect size found by Olson and Fazio [5], yielded a required sample size of *N* = 122. We recruited 123 participants for this study; three participants aborted the study, leaving 120 participants for the final analysis (Age *M* = 22.98, s.d. = 5.40). Participants were mostly University of Cologne students who received partial course credit for their participation.

#### 2.1.3. Material

Eight grey-scale drawings of cars were used as CSs. The contrast of the images were equated to ensure comparable visibility of all cars under brief presentation conditions. Large differences between images could result in high identification rates in the online measure of awareness, overestimating the visibility of the image. Based on a small pilot study (*N* = 28) the most neutral images with low standard deviations were selected as CSs for this experiment. Two additional images were selected for filler trials; 10 positive, 10 neutral and 10 negative sound files were selected from the IADS database (see appendix A [32]). All experimental scripts, data files and analysis scripts are available at https://osf.io/cx5eh/.

#### 2.1.4. Procedure

Participants were seated in front of a 60 Hz CRT monitor and instructed to attend to and memorize image-sound pairs. Furthermore, participants were told that they would be asked to identify the presented image from a set of eight images after every trial.

Each CS was randomly assigned to the positive or negative valence condition for each participant anew. CS-US pairs were shown 10 times, resulting in 80 critical trials. Each trial started with a 2100 ms blank screen, followed by a 700 ms forward mask. Afterwards, the CS was shown for either 17 ms or 1000 ms. The sound of the US had the same onset as the CS image and an average length of 3462 ms (s.d. = 359 ms). Each CS was followed by the same pixel mask for 2000 ms and a subsequent 2000 ms blank screen. The order of trials was randomized.^1^

In addition to the 80 critical trials, there were 20 filler trials in which two additional images of cars were presented for 85 ms and paired with 10 neutral sounds. These trials served the sole purpose of motivating the participants to attend to the briefly presented CSs [34]. The same two images of cars were used for these trials, and no rating data were collected for these images.

Following every critical trial, participants performed the identification task by selecting one CS from an array of all eight CSs by pressing a corresponding number on the keyboard. For filler trials, participants selected an image from an array of six random critical CSs and the two filler images. Participants were instructed to guess if they did not know which image had been presented.

After the learning task, participants indicated how much they liked each CS (presented in random order) on a slider ranging from −100 (*not at all*) to 100 (*very much*; saved as 0 to 200). In the 2-AFC task, a positively paired and a negatively paired CS from the same presentation time condition were pitted against each other at random. Each CS was shown only once in the 2-AFC, yielding four choices in total (i.e. two for CSs presented for 17 ms and two for CSs presented for 1000 ms). Participants were instructed to choose the car they would actually want to buy. The order of dependent variables was counterbalanced across participants. In the end participants provided demographic data (age, gender, and occupation), speculated about the goal of study, and could provide additional comments.

#### 2.1.5. Data analysis

We report *p*-values and Bayes factors as inference criteria for all analyses. Bayes factors are readily interpretable as changes in model odds. Thus, Bayes factors can be interpreted as a measure of evidence for an alternative hypothesis relative to the null hypothesis given the observed data [35]. We use BF_10_ to denote the evidence for the alternative hypothesis relative to the null hypothesis (i.e. BF_10_ > 1 indicates support for the alternative hypothesis) and BF_01_ to denote the evidence for the null hypothesis relative to the alternative hypothesis (i.e. BF_01_ > 1 indicates support for the null hypothesis). We report Bayes factors rather than posterior model odds because they can be used to determine the rational posterior belief in a hypothesis based on any subjective prior belief in the hypotheses. The *α*-level for all frequentist analyses was 0.05.

We performed repeated measures ANOVA to analyse evaluative ratings and paired *t*-tests to examine interaction effects. To compute ANOVA Bayes factors we used default multivariate Cauchy priors as described by Rouder and colleagues [36] with a scaling parameter of *r* = 0.5; for all *t*-tests we used Cauchy priors with a scaling parameter of $r=\sqrt{2}\text{/}2$ [37]. All Bayes factors are estimated with errors less than 1%.

To analyse 2-AFC responses we used logistic mixed effects regression models. For the frequentist analyses we specified maximal random participant effects with intercepts and slopes for presentation time [38]. We tested each effect by comparing the full model with a model without the effect of interest by means of likelihood ratio tests. For specific contrasts we compared least square means (i.e. predicted marginal means) from the full model. To compute Bayes factors we used independent Cauchy priors with scaling parameters *r* = 0.91 for all experimental factors and *r* = 1.28 for the intercept (see appendix B [39]). We chose these scaling parameters by transforming the scaling parameters used in the Bayesian ANOVA and *t*-tests to logits [40]. In this analysis the intercept corresponds to the main effect of US valence, i.e. the inclination to select the positively paired CS rather than the negatively paired CS. We chose a wider prior for the intercept in correspondence with our priors in *t*-tests on liking responses. Finally, we used the default uninformative priors specified by the *brms* package [41] for all other (nuisance) parameters. In contrast to the frequentist analysis, we added a crossed maximal random effect for CS pairs with intercepts and slopes for all within-item effects (this additional random effect was omitted in the frequentist analysis to overcome convergence problems). We estimated Bayes factors as Savage–Dickey density ratios of prior distributions and maximum-likelihood Gaussian density estimates of the posterior distributions [42].

We performed all analyses in R [43]^2^ Stan [52].

### 2.2. Results

Prior to our main analysis, we inspected correct responses in the online visibility check for CSs that had been presented for 85 or 1000 ms. We expected both stimulus durations to be long enough to allow supraliminal processing and substantially above-chance identification performance. We, thus, examined performance in these conditions to identify inattentive or unmotivated participants. With eight CS options to choose from, random guessing would yield 12.5% correct responses. Using the Tukey boxplot outlier criterion, we excluded nine participants from further analyses whose identification performance was below 12.50% at 85 ms or below 57.50% at 1000 ms.

#### 2.2.1. Visibility

The remaining 111 participants on average correctly identified 87.66% (s.d. = 32.90) of CSs presented for 1000 ms and 64.50% (s.d. = 47.86) of CSs presented for 85 ms. Correct identification for CSs presented for 17 ms was on average 21.37% (s.d. = 41.00), which was significantly above chance, *t*_110_ = 7.73, *p* < 0.001, BF_10_ > 1000, *d* = 1.75, 95% HDI [1.45, 2.05].

#### 2.2.2. Evaluation

**Figure 1.**
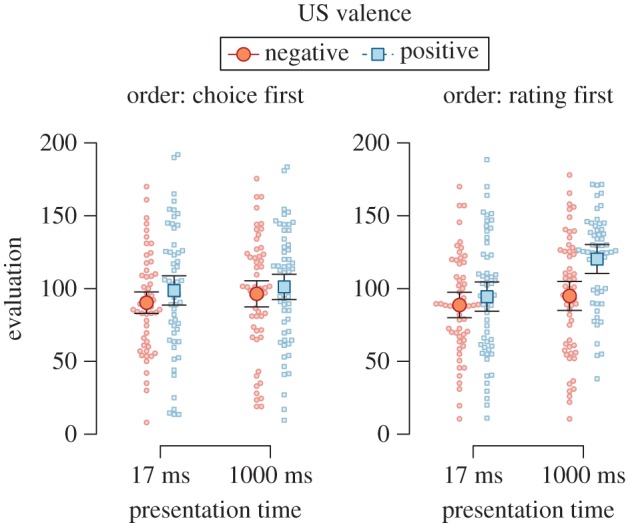
Evaluations of CSs in Experiment 1, split by the order of dependent variables, CS presentation time, and valence of the US paired with the CS. Error bars represent 95% within-subjects confidence intervals, dots represent participants' individual data points.

As can be seen in figure 1, CSs paired with positive USs were preferred to CSs paired with negative USs (BF_10_ = 28.59, *F*_1,109_ = 13.54, MSE = 1003.34, *p* < 0.001, $\eta_{G}^{2}=0.021$), and CSs presented for 1000 ms were preferred to CSs presented for 17 ms, BF_10_ = 11.88, *F*_1,109_ = 8.33, MSE = 1372.89, *p* = 0.005, $\eta_{G}^{2}=0.018$. Descriptively, there was some indication of an interaction of US valence, presentation duration, and the order of dependent variables, but the evidence was ambiguous, BF_01_ = 1.19, *F*_1,109_ = 3.13, MSE = 1189.78, *p* = 0.080, $\eta_{G}^{2}=0.006$.

We explored the data using two separate follow-up ANOVA to analyse each order-of-dependent-variables condition separately. When participants chose between CSs before rating them (i.e. choice first), we found no significant effects of our experimental manipulations on evaluative ratings (2.43 < BF_01_ < 4.61 and *p* ≥ 0.12). When participants rated CSs before choosing between them (i.e. rating first), CSs paired with positive USs were preferred to CSs paired with negative USs (BF_10_ = 25.33, *F*_1,55_ = 12.86, MSE = 1055.84, *p* = 0.001, $\eta_{G}^{2}=0.043$), and CSs presented for 1000 ms were preferred to CSs presented for 17 ms, BF_10_ = 34.71, *F*_1,55_ = 9.15, MSE = 1573.02, *p* = 0.004, $\eta_{G}^{2}=0.045$. Most importantly, the effect of US valence depended on CS presentation duration, albeit the evidence was weak, BF_10_ = 1.41, *F*_1,55_ = 4.10, MSE = 1299.90, *p* = 0.048, $\eta_{G}^{2}=0.017$. Follow-up tests did not indicate an effect of US valence when CSs were presented for 17 ms (*t*_55_ = −0.94, *p* = 0.350, BF_01_ = 2.75, *d* = 0.12, 95% HDI [−0.14, 0.37]), but showed clear indication of an effect when CSs were presented for 1000 ms, *t*_55_ = −3.73, *p* < 0.001, BF_10_ = 112.39, *d* = 0.47, 95% HDI [0.20, 0.75].

#### 2.2.3. Choice

**Figure 2.**
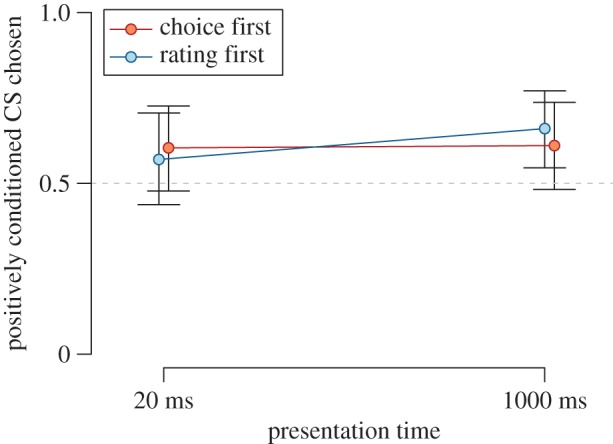
Rate of two-alternative forced choice responses between positively and negatively conditioned CSs in Experiment 1. Higher values indicate a preference for the positively conditioned CS. Points and error bars represent Bayesian estimates of condition means and the corresponding 95% highest density intervals from the logistic mixed effects regression model.

CSs that were paired with positive USs were chosen more frequently than CSs paired with negative USs in the 2-AFC task, $\chi_{1}^{2}=14.41$, *p* < 0.001, BF_10_ = 24.44, *d* = 0.25, 95% HDI [0.10, 0.41]. We found weak evidence that the inclination to choose positively paired CSs was independent of the order of dependent variables, (i.e. there was positive evidence for the absence of an effect of order; see figure 2), BF_01_ = 4.34, $\chi_{1}^{2}=0.00$, *p* = 0.979. The data did not provide evidence for or against an effect of presentation time on choice behaviour, (BF_01_ = 2.05, $\chi_{1}^{2}=1.60$, *p* = 0.206); a possible effect of presentation time appeared to be independent of the order of dependent variables, albeit the evidence was weak, BF_01_ = 3.28, $\chi_{1}^{2}=1.34$, *p* = 0.247.

We further explored the effects of CS-US pairings on choice behaviour in the condition where the 2-AFC task was administered first. Here, Bayesian and frequentist analyses yielded conflicting results. The Bayesian analysis testing whether positively paired CSs were chosen more frequently than negatively paired CSs was inconclusive, both for CSs presented for 17 ms (BF_01_ = 1.88, *d* = 0.23, 95% HDI [−0.06, 0.53]) and 1000 ms (BF_01_ = 1.67, *d* = 0.25, 95% HDI [−0.05, 0.56]). The frequentist analysis, however, indicated significant effects in both conditions, *z* = 1.85, *p* = 0.32 and *z* = 2.07, *p* = 0.019 for 17 and 1000 ms, respectively. We found no difference between the presentation time conditions, BF_01_ = 5.53, *z* = −0.09, *p* = 0.927.

### 2.3. Discussion Experiment 1

Extending the findings by Stahl and colleagues [24], we did not find evidence for automatic processes in EC in the present cross-modal procedure. For the visual CS presented for 1000 ms, our findings replicate previous studies that have demonstrated cross-modal EC.

Our results indicate that the order of our dependent variables may have affected participants' evaluative ratings of the CSs (note though that the statistical evidence was ambiguous): Only when participants evaluated CSs before choosing between them did we find an EC effect for stimuli presented for 1000 ms. Yet, irrespective of the order of dependent variables, we observed no EC effect for CSs presented for 17 ms, despite the fact that they were clearly identified better than chance. The statistical evidence of the Bayesian analyses for the absence of EC for briefly presented CSs was, however, merely suggestive. Thus, we have addressed a potential limitation of previous studies that showed the absence of an EC effect for briefly presented CSs [24], and have replicated this finding. Yet, because of the relatively weak evidence for this absence, our results need to be replicated before strong conclusions can be drawn.

The analysis of 2-AFC responses revealed an overall EC effect: Positively paired CSs were chosen more frequently than negatively paired CSs. This result indicates that the implemented 2-AFC task in which participants choose between positively and negatively paired CSs is suitable to assess EC. The preference for positively paired CSs appeared to be independent of the presentation time of CSs during the learning phase and the order in which the dependent variables were measured. However, when exploring choice responses of participants who performed the choice task before evaluating CSs, our data, again, did not provide conclusive evidence. Bayesian and frequentist analyses yielded conflicting results and, thus, should be interpreted cautiously. Nevertheless, the 2-AFC task appeared to be a valid measure of EC. A replication in a larger sample could shed more light onto the possibility that choice might be more sensitive than evaluative rating to detect potentially subtle EC effects for briefly presented CSs.

#### 2.3.1. Limitations of Experiment 1

Our findings challenge the existence of an automatic associative conditioning process underlying EC. One might, however, criticize the continuous assessment of CS visibility during the learning task. Whereas the visibility check provides strict control over awareness of CSs, it could be argued to interfere with the automatic conditioning process. Specifically, the visibility check may induce highly focused and deliberative stimulus processing and thus obscure the influence of unaware learning experiences. The deliberative and analytic mindset may have been further promoted by the instructions to memorize the CS-US pairs. In short, it is possible that our visibility check and instructions obstructed the automatic EC process.

Three additional aspects of our experimental design may constitute suboptimal conditions for obtaining an automatic EC effect for briefly presented CSs. First, our CSs were highly similar in shape and contrast to ensure equivalent visibility under brief viewing conditions. Similar stimuli may result in a confusability that could affect automatic associative processing of the CSs. Thus, the high similarity between briefly presented CSs may mitigate a potentially weak EC effect.

Second, the duration of USs used in Experiment 1 was relatively long (*M* = 3462 ms, s.d. = 359 ms) compared to the briefly presented CS. This mismatch in presentation duration may have resulted in a higher salience of the US compared to the CS, which, as suggested by Jones and colleagues [53], might have inhibited automatic EC effects. Third, a simultaneous presentation onset of CS and US might not have resulted in a simultaneous processing of both stimuli: Specifically, the valence of the auditory US may have been extracted only after a delay that may have been longer than the entire presentation duration of the CS, such that the processing of the affective response may have been delayed to after the processing of the CS.

To sum up the findings of Experiment 1, while the paradigm is well suited to study EC, it might not yet provide optimal conditions for automatic processes in EC by way of implicit misattribution. Thus, the results of Experiment 1 can be considered inconclusive for several reasons. In Experiments 2 and 3, we aimed at replicating and extend our findings to address methodological criticism.

## 3. Unregistered Experiment 2

In Experiment 2, we used different materials and modified several aspects of the procedure. Experiment 2 aimed at replicating the pattern of EC effects across CS presentation-duration levels on evaluative ratings as the sole dependent measure. Experiment 2 also implemented an online CS identification task; data from this task was to serve as reference for a final Experiment 3 in which the online CS identification task was omitted.

We used new CSs for which mean visibility under the presentation conditions was unknown, so we again used an online visibility check to estimate CS identification performance for these new CSs across critical presentation-duration levels, and to find presentation conditions under which the new CSs were identified at slightly above-chance levels. Based on previous findings from our laboratory [24], we selected duration levels of 20 and 30 ms, in addition to a 1000 ms condition.

Furthermore, we compared the simultaneous CS-US onset, which we used in Experiment 1, to a 400 ms delayed onset of the CS relative to the US. Using this onset asynchrony, the briefly presented CS was presented in the middle of the auditory US in order to create a stronger sense of simultaneous CS and US processing as compared to a mere simultaneous CS-US presentation onset. More specifically, we estimate that the affective response to be misattributed to the CS according to the implicit-misattribution account may take approximately 400 ms to develop: Research has shown that the pupillometric response to a valent sound starts after 400 ms [54], indicating that the valence of the sound is actively processed after this interval. By using a 400 ms stimulus onset asynchrony (SOA), we ensured that the CSs presented during that time are especially likely to be the target of implicit misattribution, and therefore, likely to show an automatic EC effect.

### 3.1. Method

#### 3.1.1. Design

In Experiment 2 we manipulated the presentation time of the CS (20 ms versus 30 ms versus 1000 ms) as well as the valence of the US (positive versus negative) within participants. Half of the CSs presented for 1000 ms had a simultaneous CS-US onset, the onset was delayed by 400 ms for the other half, and all CSs presented for 20 or 30 ms were delayed by 400 ms. We assumed that a delayed onset would be better suited to induce a simultaneous experience of CS and US when the CS is presented briefly. To test our intuition and ensure that a delayed onset yields an EC effect of at least the same magnitude, we compared the delayed and simultaneous onset in the long CS presentation condition. To focus our resources on the more critical evaluative ratings measure, we did not administer the 2-AFC task in Experiment 2.

#### 3.1.2. Material

##### 3.1.2.1. Conditioned stimulus

As CSs, we used Pokémon cartoons similar to those used in previous studies [55]. The images were changed to greyscale and contrasts were slightly reduced. Twenty-four stimuli were pretested in a small pilot study (*N* = 27). Eight cartoons were selected based on mean evaluative ratings within 5% above or below the mean of the scale and the lowest standard deviation. The selected set had a mean evaluative rating of 98.69 and standard deviation of 36.33 on a 201 point slider scale (see Experiment 1).

For each participant, two different CSs were shown for 20 ms, for 30 ms, for 1000 ms with a delayed CS-US onset, and for 1000 ms with a simultaneous CS-US onset. Each CS was randomly assigned to one of the four conditions for each participant anew.

##### 3.1.2.2. Unconditioned stimulus

We used a set of 10 harmonious and 10 disharmonious tones [56,57], which were cut into 800 ms segments, as USs. Eight of these tones have been successfully used in a previous cross-modal EC study [58] and we selected 12 additional similar tones from the set. Finally, we created 10 neutral sounds that consisted of three metronome ticks in varying pitches.

#### 3.1.3. Procedure

Participants were seated in front of a 100 Hz CRT monitor. In contrast to Experiment 1, participants were not instructed to memorize CS-US pairs. They were instructed to pay attention to the sounds and images, and told that they would be asked to identify the images after every trial.

Each trial started with a blank screen for 500 ms, followed by a fixation cross shown for 500 ms. Then a black-and-white pixel mask was shown for 500 ms (which was randomly rotated by 0, 90, 180 or 270 degrees), followed by the CS presentation (duration was 20 ms, 30 ms, 1000 ms, or 80 ms for filler trials as in Experiment 1). As mentioned above, US presentation started 400 ms before the onset of the CS in the 20 ms, 30 ms, and half of the 1000 ms trials. For the other half of the 1000 ms trials, the onset of CS and US was simultaneous. The CS was immediately followed by a post-mask shown for 100 ms (which was again randomly rotated by 0, 90, 180 or 270 degrees, but at a different angle than the forward mask). Afterwards a blank screen was shown for 1000 ms.

Similar to Experiment 1 we included a visibility check after every trial. However, in Experiment 2 we gave participants only CSs that were shown for a similar presentation time (either for 20 ms and 30 ms or for 1000 ms) to choose from, in order to better control for guessing strategies. Participants may have correctly reasoned that a CS that was previously presented and correctly perceived at a longer presentation time may not be a plausible option in trials with a brief presentation time (such guessing strategies may have inflated previous estimates of stimulus visibility). Participants were again asked to select the CS they had seen during the trial from a list of these four CSs. If the CS was shown for 80 ms, two additional Pokémon images that were not used in the learning phase were given as distractors.

After evaluating the critical CSs as in Experiment 1, participants indicated how well they knew the Pokémon on a 4-point scale (*not at all*, *looks familiar*, *know it*, *know it very well*) and answered the same demographic questions as in Experiment 1. Additionally, we asked participants whether they had worn the headphones during the entire procedure.

#### 3.1.4. Sample

We used a sequential Bayesian analysis to analyse the incoming data [59]. We started analysing the data after collecting 30 participants to reduce the chance of false positive evidence [60] and analysed the data after every day of data collection. Our main analyses consisted of two *t*-tests for EC effects in the 1000 ms condition separately for the simultaneous and delayed CS-US onset. We planned to collect data until the Bayes factor for both EC effects was larger than 5 or until a maximum of 55 participants was collected.

These criteria were met, and data collection was therefore stopped, after 46 participants. Participants were mostly University of Cologne students and received partial course credit or €2 for their participation. Five participants were excluded due to poor performance on the visibility check at 80 and 1000 ms (see Experiment 1 for details). Three additional participants were excluded because they reported that they did not wear the headphones during the entire procedure. Two additional participants reported for at least one CS, that they knew it very well and were therefore excluded. We, thus, included 37 participants in the final analysis (Age *M* = 23.51, s.d. = 3.83, 26 female).

#### 3.1.5. Data analysis

We used Bayes factors for inference in all analyses as described in Experiment 1. We again performed repeated-measures ANOVA to analyse liking responses and paired *t*-tests to examine interaction effects. We only report Bayesian analyses because *p*-values are uninterpretable when the sampling plan is not clearly defined *a priori*, as was the case in our design [61].

### 3.2. Results

#### 3.2.1. Visibility

One goal of Experiment 2 was to ensure that the new stimulus material was identified at slightly above-chance levels. Participants were asked to identify the CS from an array of four CSs, which resulted in a chance level of 0.25. One stimulus was correctly identified in 90% of cases in the 20 ms condition and in 93.33% of cases in the 30 ms condition. We excluded this stimulus for all further visibility analyses. The average correct identification rate in the 20 ms condition was 0.45, ranging from 0.36 to 0.54, which was clearly above chance, BF_10_ = 932.30, *d* = 1.77, 95% HDI [1.25, 2.32]. The CSs showed highly similar standard deviations, ranging from 0.48 to 0.50. Similarly, in the 30 ms condition the average correct identification rate was 0.57, ranging from 0.46 to 0.80, which was significantly above chance, BF_10_ > 1000, *d* = 2.21, 95% HDI [1.61, 2.85] (standard deviations in the 30 ms condition ranged from 0.40 to 0.50).^3^

#### 3.2.2. Evaluation

**Figure 3.**
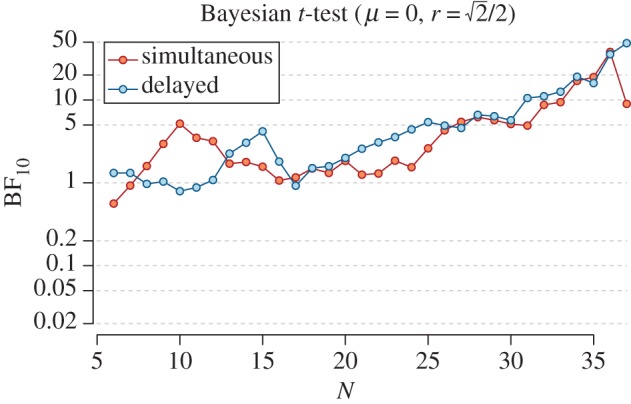
Sequential Bayes factor of *t*-tests of the 1000 ms condition in Experiment 2 (with the visualization starting after five participants).

As shown in figure 3, Bayes factors for the simultaneous (BF_10_ = 9.86, *d* = 0.43, 95% HDI [0.10, 0.76]) and delayed CS-US onset (BF_10_ = 49.94, *d* = 0.54, 95% HDI [0.20, 0.89]) were larger than 5, which was our stopping rule, and both developed similarly over the course of data collection. The data, moreover, indicated that there was no difference between the delayed and simultaneous onset in the EC effect for CSs presented for 1000 ms, BF_01_ = 3.95, $\eta_{G}^{2}=0.003$.

**Figure 4.**
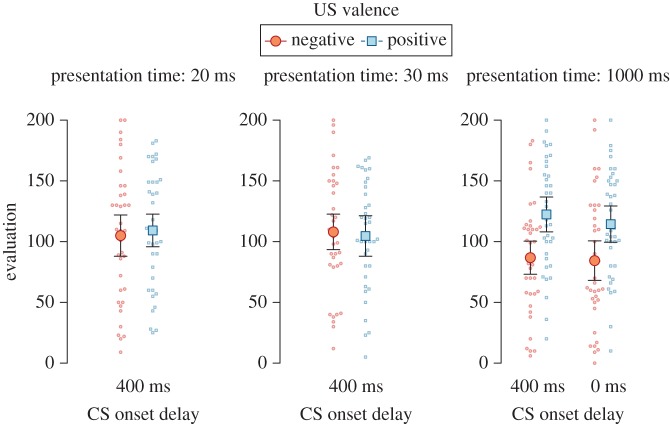
Evaluations of CSs in Experiment 2, split by the presentation time (20, 30 or 1000 ms) and the SOA of CS and US (delayed onset by 400 ms of the CS or simultaneous onset of CS and US for one 1000 ms condition only). Error bars represent 95% within-subjects confidence intervals, dots represent participants' individual data points.

We computed an ANOVA for presentation time (20, 30, 1000 ms) × US valence (positive, negative) and found evidence for an interaction between the two factors, BF_10_ = 5.17, $\eta_{G}^{2}=0.029$, see also figure 4. As in Experiment 1, we found an EC effect only for CSs presented for 1000 ms and not for briefly presented CSs: There was a robust EC effect only for CSs presented for 1000 ms, BF_10_ = 394.81, *d* = 0.66, 95% HDI [0.31, 1.03]. In contrast, there was some evidence for the absence of an EC effect for CSs presented for 20 ms, BF_01_ = 4.12, *d* = 0.06, 95% HDI [−0.25, 0.37], as well as for CSs presented for 30 ms, BF_01_ = 7.01, *d* = −0.04, 95% HDI [−0.35, 0.27].

### 3.3. Discussion Experiment 2

Replicating Experiment 1 with different CS and US materials, Experiment 2 found a cross-modal EC effect for CSs presented for 1000 ms, but not for those presented for 20 or 30 ms. Additionally, there was no detrimental effect of the 400 ms SOA on EC for supraliminal CSs: Based on the findings of Experiment 2, we conclude that a slight CS-US onset asynchrony does not affect the EC effect at long presentation times compared to a simultaneous CS-US onset. We speculate that using a 400 ms delayed CS-US onset may induce a stronger experience of simultaneous presentation for briefly presented CSs compared to a simultaneous onset. We propose that the delayed CS-US onset may provide more favourable conditions for EC effects with briefly presented CSs, as it might increase the chances of affective responses to the US being experienced simultaneously with the CS presentation.

The visibility of the briefly presented CSs was clearly above chance even for stimuli presented for 20 ms. This was expected, given that the CSs used in Experiment 2 were more easily discriminable than those used in Experiment 1, and given that CS duration was slightly longer than the 17 ms implemented in Experiment 1. CSs were identified above chance, but the correct identification rates were much lower than for stimuli presented for 80 or 1000 ms. Presenting the CSs for 20 ms therefore yields a suboptimal yet clearly not invisible method of presentation given the stimuli and procedure at hand.

Finally, there was no indication for an EC effect for briefly presented CSs. Both in the 20 ms and in the 30 ms condition, the data suggested that a near-threshold EC effect was absent. Although we made procedural changes to create more favourable conditions for automatic processes in EC compared to Experiment 1 (i.e. we eliminated instructions to memorize CS-US pairs, high similarity between CSs, and simultaneous CS-US onsets), we were not able to find an EC effect with briefly presented CSs. But, again, caution is warranted when drawing conclusions about automatic processes in EC based on the findings of Experiment 2 alone. The evidence for the absence of an EC effect was moderate and, as mentioned above, we cannot preclude that the online visibility check interfered with an automatic processing of the pairings. We therefore aimed at a replication of Experiment 2 with a larger sample and including again the potentially more sensitive 2-AFC measure.

## 4. Registered Experiment 3

The goal of Experiment 3 was to replicate the findings of Experiments 1 and 2 in a sample large enough to detect medium to small EC effects for near-threshold CSs, or conversely to accumulate conclusive statistical evidence for the absence of such an effect. The only major difference to Experiment 2 was that we did not administer the visibility check throughout the learning task to avoid inducing an analytic deliberative mindset that may have obstructed automatic processes in Experiments 1 and 2 [24]. As in Experiment 1, we additionally assessed CS preferences in a 2-AFC choice task. In a high-powered study, this inclusion will contribute to answering the question whether choice is a more sensitive measure to detect small changes in preference as compared to evaluative ratings. From the visibility data of Experiment 2, we can conclude that the visibility of the briefly presented CSs was above chance even at 20 ms. Given that CSs are identified above chance, an absence of an EC effect for briefly presented CSs would be highly informative because we have attempted to create optimal conditions under which dual-process theories of EC would predict an automatic EC effect.

If, on the other hand, we observed an EC effect with briefly presented CSs, the results would contradict findings by Stahl and colleagues [24] who found no EC effect for near-threshold but above-chance visual CSs. Such a result would call into question their conclusion that there is no automatic EC effect, and it would lend credibility to the critique that the absence of EC in previous studies may have been caused by the experienced delay between CS and US, by a focused, analytic stimulus-processing mindset induced by instructions to memorize CS-US pairings, or by interference from the online visibility check. Thus, Experiment 3 will provide a stringent and fair test of the predictions of single- versus dual-process models of EC.

### 4.1. Procedure

The procedure was the same as in Experiment 2 except that all briefly presented CSs were displayed for 20 ms and all CS onsets were delayed by 400 ms relative to USs. The study, thus, followed a 2 (*presentation time*: 20 or 1000 ms) × 2 (*US valence*: positive or negative) × 2 (*DV order*: 2-AFC or rating first) design; the first two factors were manipulated within participants. The order of dependent variables was counterbalanced across participants.

We substituted the visibility task with a target-detection task similar to the one used by Olson & Fazio [5] in the surveillance paradigm. In Experiment 3, one of the two filler CSs was randomly selected to serve as the target stimulus for this task. It was presented for 200 ms (while the other filler stimulus was presented for 80 ms), and participants were instructed to press the space bar as quickly as possible whenever it appeared on screen during the learning phase. This task achieves the goal of focusing participants' visual attention on the CSs in a manner comparable to the CS identification task while being much less resource-demanding and considerably easier to perform. Most importantly, the task has repeatedly been shown to successfully support EC effects in incidental EC procedures that are supposedly due to the operation of automatic associative processes [5].

#### 4.1.1. Material

Based on the combined visibility data from Experiment 2 and the small pilot test administered afterwards, we decided to select the four most clearly visible CSs to be shown for 1000 ms only and the four CSs with lowest visibility to be presented for 20 ms only (for an overview of the visibility rates see appendix C). Note that all CSs were identified at above-chance levels in both datasets.

### 4.2. Analysis plan

Before conducting our analyses, we excluded participants who (i) reported for at least one of the Pokémon CSs that they ‘know it very well’ (because pre-experimental preferences towards the stimuli are typically not affected by EC); (ii) reported that they did not wear the headphones during the learning task; (iii) missed too many targets in the ongoing task (participants were excluded based on Tukey's outlier criterion, but only if that criterion reaches 3 misses, thereby allowing for 1 or 2 lapses during the learning phase); (iv) aborted the experiment prematurely; or (v) explicitly reported other major (but unforeseeable) issues during the experiment that hindered them from participating in an orderly fashion (e.g. loud noises during experiment, difficulty understanding the instructions; based on our experience from previous studies we expect at most one of such cases to occur).

After the initially set *N* was reached, we analysed the data using frequentist and Bayesian data analyses. All further incoming data were analysed using a sequential Bayesian analysis [59,60].

#### 4.2.1. Planned sample size

We first collected a fixed number of 122 participants based on the same *a priori* power analysis as described for Experiment 1, which ensured adequate power for a one-sided paired *t*-test with *α* = *β* = 0.05 and medium-to-small effects of *d* = 0.3 that are at the lower end of typical incidental EC effect sizes. A minimal *N* = 122 should also help to minimize the chance of a false positive finding in the sequential Bayesian analyses [60]. We continued data collection until all targeted Bayes factors had reached (or exceeded) 10 or greater (or less than 1/10 when the inverse Bayes factor is considered), or until we ran out of money (i.e. participants were paid €2 for the short study, and funds were available to pay a maximum of *N* = 300 participants). The total *N* might be higher than 300 because some participants may opt to participate in exchange for partial course credit. After the minimal sample size was reached, the data were analysed after every day of data collection.

##### 4.2.1.1. Stopping rule

We planned to stop data collection when the evidence for both EC effects (20 and 1000 ms) was conclusive, both for evaluative ratings and the choice measure. That is, we monitored four Bayes factors, and we stopped data collection when, in each of the four cases, either BF_01_ > 10 or BF_10_ > 10.

#### 4.2.2. Confirmatory analysis

In our confirmatory Bayesian analyses, which were also the basis of our data collection stopping rule, we first tested if the order of dependent measures affects the outcome of our experimental manipulations. Only when finding compelling evidence that the order of dependent measures did not affect results (BF_01_ > 10) we ignored the factor of dependent measure order. If we observed inconclusive statistical evidence, or if we found evidence that the order of dependent measures affected our results (BF_10_ > 10), we would analyse the evaluation data only for the subset of participants who first evaluated the CSs, and vice versa for the choice data analysis. For the frequentist analyses planned to be conducted on the initial fixed sample size of *N* = 122, we would ignore the factor of dependent measure order if we did not find a significant effect of the order on our results.

### 4.3. Initial confirmatory analysis

To probe for the presence or absence of EC effects within each level of the presentation time factor (20 and 1000 ms), we calculated one-tailed (Bayesian and frequentist) paired *t*-tests. We used the same priors and settings as in Experiments 1 and 2. For the initial analysis, we used the first 122 valid datasets (i.e. continued data collection if we had to exclude participants for any of the reasons given above until *N* = 122 was reached).

#### 4.3.1. Evaluation

Since the results of the frequentist and Bayesian analyses diverged, we will report them separately.

##### 4.3.1.1. Frequentist analysis

There were no indications for an interaction of the measurement order and the US valence, *F*_1,120_ = 2.47, MSE = 1174.34, *p* = 0.118, $\eta_{G}^{2}=0.004$, nor the measurement order and the US valence and CS presentation time, *F*_1,120_ = 0.63, MSE = 1335.92, *p* = 0.429, $\eta_{G}^{2}=0.001$. We therefore used the data of all participants (i.e. participants who did the evaluation task first and participants who did the choice task first) for further analyses of the evaluation. Of the CSs presented for 1000 ms, those shown with positive USs were evaluated more positively than those presented together with negative USs, *t*_121_ = 4.72, *p* < 0.001, *d* = 0.43. There were no indications for an EC effect for CSs presented for 20 ms, *t*_121_ = 0.51, *p* = 0.307, *d* = 0.05.

##### 4.3.1.2. Bayesian analysis

The Bayesian analyses on the potential influence of the order of measurement did not yield sufficient evidence to ignore the factor of measurement order. This was true for the three-way interaction of order × presentation time × US valence, BF_01_ = 3.99, $\eta_{G}^{2}=0.001$, and the interaction between US valence and order, BF_01_ = 2.58, $\eta_{G}^{2}=0.004$. For the following analyses, we therefore only used the data of participants who performed the evaluation task before the choice task (*N* = 59). As in the frequentist analyses, for the CSs presented for 1000 ms a clear EC effect was found, BF_10_ = 498.39, *d* = 0.53, 95% HDI [0.26, 0.80]. There was however, no evidence for nor against an EC effect for CSs presented for 20 ms, BF_10_ = 1.20, *d* = 0.22, 95% HDI [−0.03, 0.47]. Since both of these Bayes factors were part of the sequential stopping rule, we continued data collection after the initial data analysis.

#### 4.3.2. Choice

We analysed 2-AFC responses using logistic mixed effects regression models, using the same specifications as in Experiment 1 for both frequentist and Bayesian analyses. In these analyses, in which the proportion of choices of the CS paired with positive USs is the dependent variable, the intercept can be interpreted as a main effect of US valence, corresponding to an inclination to select positively paired CSs over negatively paired CSs. A main effect of presentation time would be equivalent to a modulating effect on EC, indicative of a stronger EC effect for one of the two levels of the presentation-duration factor. We planned to assess the respective intercept terms to probe for the presence of EC effects on choice in each level of the presentation-duration factor.

##### 4.3.2.1. Frequentist analysis

No significant interaction of order and US valence, $\chi_{1}^{2}=0.03$, *p* = 0.858, nor a significant three-way interaction of the order of dependent measures × US valence × presentation time of the CS was found, $\chi_{1}^{2}=0.36$, *p* = 0.549. We therefore ignored the order factor and analysed the data of all participants. Of the CSs presented for 1000 ms, those conditioned with positive USs were chosen more often than those paired with negative USs, *z* = 3.87, *p* < 0.001. For CSs presented for 20 ms, there was no indication for a preference for CSs paired with positive USs, *z* = −0.41, *p* = 0.685. The results of the choice variable therefore reflected the result of the evaluation variable reported above.

##### 4.3.2.2. Bayesian analysis

Similar to the analyses of the evaluation data, the Bayes factors for the interaction of the US valence and the order of dependent measures, BF_01_ = 4.40, as well as the interaction of US valence × order of dependent variables × CS presentation time, BF_01_ = 3.88, did not reach our pre-set level of a Bayes factor larger 10 for the null hypothesis, which would have allowed us to ignore the order of dependent variables. We therefore only analysed the data of participants who performed the choice task before the rating task (*N* = 63). Even though, descriptively, CSs shown for 1000 ms and conditioned with positive USs were more likely to be selected, the Bayes factor was inconclusive, BF_10_ = 2.64, *d* = 0.37, 95% HDI [0.06, 0.69]. The Bayes factor was more conclusive for stimuli presented for 20 ms: The data supported the null hypothesis of no preference for CSs conditioned with positive USs, BF_01_ = 12.01, *d* = 0.03, 95% HDI [−0.23, 0.28].

Since we did not find conclusive evidence for all four target Bayes factors in our initial analysis, we continued data collection and analysed the data after every day of data collection.

### 4.4. Confirmatory analyses after sequential testing

During the sequential data collection, one of the target Bayes factors did not reach our pre-set level (i.e. >10 or <1/10), and we therefore collected the previously set maximum data of 300 paid participants (363 participants in total, including participants who took part for partial course credit). Two participants took part twice and the second dataset was removed. The data of one participant was removed because she reported that she did not wear the headphones and two participants were excluded due to technical difficulties. Additionally, 42 participants were excluded because they did not press the space bar at least eight times when the target was shown and additional 56 participants were excluded because they reported to know at least one Pokémon very well. Consequently, 260 participants are included in the analysis (age *M* = 23.58, s.d. = 6.03; 208 female).

#### 4.4.1. Evaluation

**Figure 5.**
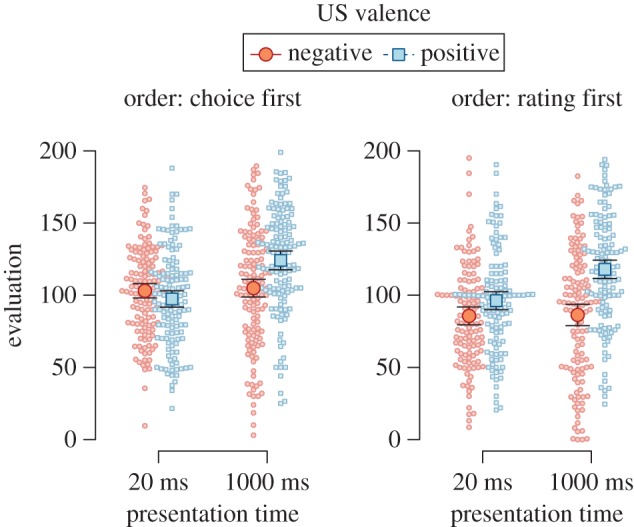
Evaluations of CSs in Experiment 3, split by the order of dependent variables, CS presentation time, and valence of the US paired with the CS. Error bars represent 95% within-subjects confidence intervals, dots represent participants' individual data points.

**Figure 6.**
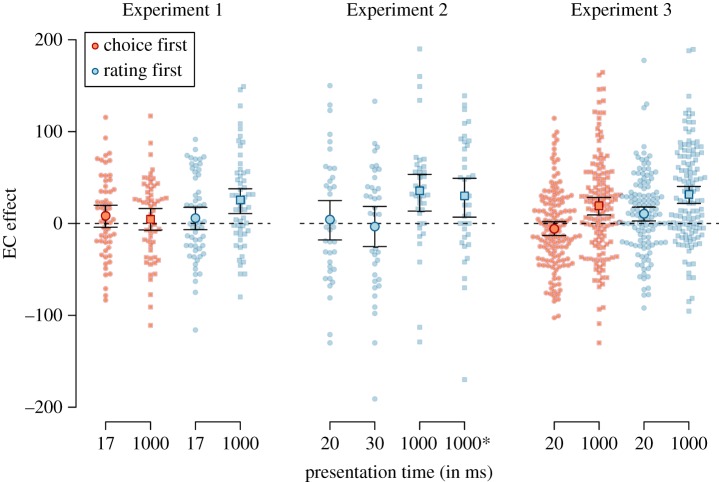
EC effects from all three experiments in the evaluative rating, split by the order of dependent variables and CS presentation time. Values above 0 indicate a more positive evaluation of CSs paired with positive USs than paired with negative USs. Large dots represent the mean EC effect and the corresponding error bars the 95% highest density intervals. Small dots represent participants' individual EC effects. * = simultaneous CS-US onset.

Since we observed an interaction between US valence and the order of dependent measures, BF_10_ = 17.74, $\eta_{G}^{2}=0.009$, we analysed only the data of participants who performed the evaluation task before the choice task (*N* = 125). As in the initial analysis, we observed an EC effect for CSs presented for 1000 ms, BF_10_ > 1000, *d* = 0.59, 95% HDI [0.40, 0.78]. Interestingly, we also observed an indication for the presence of an EC effect for briefly presented CSs, BF_10_ = 6.88, *d* = 0.24, 95% HDI [0.06, 0.41], see figure 5 (and see figure 6 for a visual comparison of all EC effects). Even though this Bayes factor did not reach our pre-set thresholds, and the effect size is small, the results indicate that even when CSs were presented for only 20 ms, participants evaluated CSs presented with positive USs as more positive than CSs presented with negative USs.

#### 4.4.2. Choice

**Figure 7.**
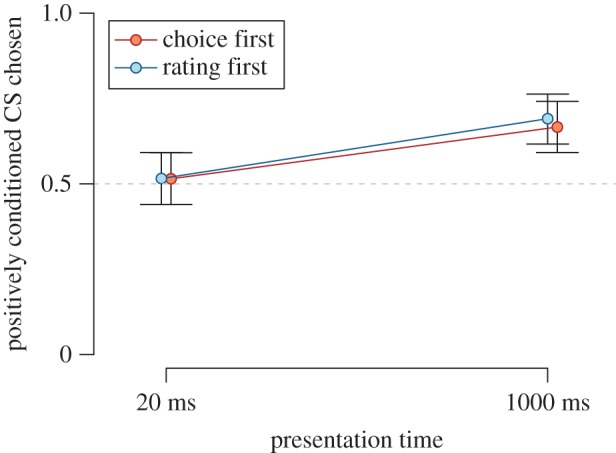
Rate of two-alternative forced choice responses between positively and negatively conditioned CS in Experiment 3. Higher values indicate a preference for the positively conditioned CS. Points and error bars represent Bayesian estimates of condition means and the corresponding 95% HDI from the logistic mixed effects regression model.

As with the initial analysis, we did not find sufficient evidence—according to our pre-set thresholds—for the absence of an influence of the order of dependent measures (Order × US valence: BF_01_ = 6.68, *d* = −0.02, 95% HDI [−0.10, 0.07]; Order × US valence × CS presentation time: BF_01_ = 6.70, *d* = 0.02, 95% HDI [−0.07, 0.10]) and therefore only analysed the participants who completed the choice task before the evaluation task (*N* = 135). After the sequential analysis, we observed statistical evidence for a preference for CSs conditioned with positive USs, when the CSs were presented for 1000 ms, BF_10_ = 339.31, *d* = 0.38, 95% HDI [0.20, 0.57]. When the CSs were presented for 20 ms, we found evidence for the absence of a preference for CSs conditioned with positive USs, BF_01_ = 15.12, *d* = 0.03, 95% HDI [−0.13, 0.20], see figure 7. Note that the EC effects on the choice variable were consistently smaller than the EC effect on the evaluation variable.

### 4.5. Exploratory analysis

In a first set of additional—not registered—analyses, we explored the sensitivity of our results to our pre-registered participant exclusion criteria. In a second set of analyses, we explored the interaction between our two dependent variables.

#### 4.5.1. Different participant exclusion criteria

In a first analysis we focused on the small EC effect on evaluative ratings obtained for CSs presented for 20 ms; this effect was based on the subset of participants who completed the evaluation task before the choice task. Here, we again analysed only the evaluation data for those participants, and we again excluded all participants who did not press the space bar at least eight times. The only difference to our confirmatory analyses was that we only excluded those participants who reported to know any of the *Pokémons shown for 20 ms* very well (in the confirmatory analysis, a participant was excluded as soon as she indicated knowing *any* Pokémon very well). In effect, this analysis included 137 participants (instead of 125 in the confirmatory analysis). Including participants who report to know any of the Pokémon shown for 1000 ms very well—but none of the Pokémon presented for 20 ms—should not have an effect on the evaluation of CSs in the 20 ms condition. However, the results of the evaluation analysis for CSs presented for 20 ms (evaluation-first only) was now inconclusive, BF_10_ = 1.40, *d* = 0.17, 95% HDI [0.01, 0.34] (i.e. there was no evidence for a preference for positively paired CSs over negatively paired CSs any more). Even though adding these participants to the analysis should not influence the ratings for CSs presented for 20 ms, the Bayes factor was reduced and we did not find any statistical evidence for the alternative hypothesis over the null hypothesis.

We additionally explored the effect of relaxing all previously set exclusion criteria (i.e. include participants who did not press the space bar often enough, include participants who knew at least one of the Pokémon very well, or both) on the choice measure as well as the evaluation measure (see table 1), with the following results: For the choice measure, relaxing any of the exclusion criteria did not affect the pattern of results. Similarly, the conclusions remain unaltered for EC effects on evaluative ratings of CSs presented for 1000 ms. Results changed only for evaluation of CSs presented for 20 ms; here, the Bayes factor was smaller—and inconclusive—when additional participants (other than those in the registered analysis) were included. It should, however, be noted that, in these analyses, we also found no statistical evidence for the absence of an EC effect for CSs presented for 20 ms.

**Table 1.** Effect of different exclusion criteria on the evaluation (evaluation first) and choice (choice first) dependent variables. In the ‘include space’ analyses only participants who reported to know at least one of the Pokémon very well were excluded. In the ‘include knowing’ analyses only participants who did not press the space bar at least eight times were excluded.

presentation time	registered	include space	include knowing	include both
evaluation				
1000 ms BF_10_	>1000	>1000	>1000	>1000
20 ms BF_10_	6.88	2.77	2.45	1.48
*N*	125	144	154	179
choice				
1000 ms BF_10_	339.31	>1000	>1000	>1000
20 ms BF_10_	1/15.12	1/14.66	1/19.01	1/18.16
*N*	135	149	162	179

#### 4.5.2. Influence of order of the dependent variables

##### 4.5.2.1. Choice

First we checked whether the choice results were affected by the order of dependent measures. We analysed the data of the evaluation-first group and found evidence for the absence of an EC effect in the 20 ms condition, BF_01_ = 13.78, *d* = 0.06, 95% HDI [0.04, 0.09], but evidence for EC in the 1000 ms condition, BF_10_ > 1000, *d* = 0.43, 95% HDI [0.25, 0.62]. This pattern of results was equivalent to the choice-first group. Unsurprisingly, when we analysed the entire sample and ignored the order of dependent variables, we found evidence for the absence of an EC effect in the 20 ms condition, BF_01_=18.45, *d* = 0.03, 95% HDI [−0.09, 0.15], but evidence for an EC effect in the 1000 ms condition, BF_10_ > 1000, *d* = 0.41, 95% HDI [0.27, 0.55].

##### 4.5.2.2. Evaluation

As reported above, we observed a clear indication for an interaction between US valence and the order of dependent measures in the evaluation task, BF_10_ = 17.74, $\eta_{G}^{2}=0.009$. There was an indication for the absence of the interaction between US valence, the order of dependent measures, and the presentation time of CSs, BF_01_ = 6.81, $\eta_{G}^{2}=0.000$. Interestingly, for the CSs shown for 1000 ms the interaction between US valence and order of dependent variables was inconclusive, BF_01_ = 1.37, $\eta_{G}^{2}=0.006$, but we found clear indications for an US valence × order of dependent variables interaction for CSs presented for 20 ms, BF_10_ = 12.08, $\eta_{G}^{2}=0.015$. This interaction for CSs presented for 20 ms reflects the finding that the EC effect was larger when the evaluation was administered before the choice task, than when it was administered after the choice task, BF_10_ = 9.91, *d* = 0.36, 95% HDI [0.11, 0.60] (a parallel comparison for CSs presented for 1000 ms yielded inconclusive results, BF_01_ = 1.48, *d* = 0.22, 95% HDI [−0.02, 0.45]).

### 4.6. Discussion Experiment 3

The main goal of the third experiment was to investigate whether we can find EC effects with brief (20 ms) and longer (1000 ms) CS presentation times when participants do not have to answer to a visibility check after every trial. For an evaluation of CSs after the learning phase as well as a 2-AFC task, we found that CSs presented for 1000 ms and paired with positive USs were preferred over CSs presented for 1000 ms and paired with negative USs. On the evaluative rating measure, we also found some evidence that CSs presented for 20 ms and paired with positive USs were rated as more positive than CSs presented for 20 ms and paired with negative USs. Interestingly, this pattern was not found in the choice task: Here, CSs presented for 20 ms were as likely to be chosen when they were paired with positive USs as when they were paired with negative USs. We additionally observed that the EC effect on evaluative ratings in the 20 ms condition was modulated by the choice task, in that the EC effect was smaller after the choice task compared to the effect found when the evaluation was administered before the choice task. In the following section, we will discuss the finding of an EC effect for CSs presented for 1000 ms, the diverging findings of EC effects for CSs presented for 20 ms, and the potential interference of the choice task on the rating task.

The finding of an EC effect in the evaluation of CSs presented for 1000 ms replicated the previous two experiments, as well as previous studies on cross-modal EC with stimuli that can be consciously perceived. The result therefore shows that the present paradigm is capable of reliably demonstrating cross-modal EC effects in evaluative ratings. Results were somewhat more mixed with regard to the choice measure: In Experiment 1, the evidence for an EC effect for CSs presented for 1000 ms in the 2-AFC task was not conclusive. In Experiment 3, however, we found compelling evidence that CSs presented for 1000 ms and paired with positive USs were preferred over CSs presented for 1000 ms and paired with negative USs. This finding shows that evaluative conditioning might be a useful tool to influence decision-making behaviour, and it lends ecological validity to the EC phenomenon. Future studies in an applied setting could further build on this finding.

In contrast to CSs presented for 1000 ms, we did not find a preference for positively paired CSs presented for 20 ms in the 2-AFC task. We can only speculate whether this is due to the fact that either it is simply not possible to influence participants' decision-making behaviour with briefly presented CSs, or whether the choice task was not sufficiently sensitive to detect small effects. Based on previous findings [31], we had originally speculated that choice might be a more sensitive measure of preferences than evaluations. In both Experiment 1 and Experiment 3—which used both choice and evaluation as dependent measures—effect sizes in the evaluation task were larger than effect sizes in the choice task. In light of these findings, we would argue that choice might not be a more sensitive measure than evaluation.

Consistent with this interpretation, there was some evidence for an EC effect in the 20 ms condition in the evaluation (but not the choice) task. For the interpretation of this effect, it should be noted that—as in the study by Stahl and colleagues [24]—the briefly presented CS stimuli were not truly subliminal but in fact visible at above-chance levels. We therefore refrain from claiming that we have found an EC effect for stimuli that were truly subliminal. Nevertheless, the present indication for an EC effect for stimuli presented only very briefly contradicts previous findings [24] suggesting that EC effects require much longer CS presentation durations. There are a few factors that might explain this discrepancy: In contrast to the experiments by Stahl and colleagues [24], the present studies (i) did not include a visibility check after each trial, and (ii) they implemented a cross-modal EC procedure which might have allowed for a more simultaneous experience of CS and US [26]. Combined, these changes might have resulted in the formation of an EC effect even with a very brief presentation time. It has to be noted, however, that the effect size found in this study was very small; this could explain why previous studies (e.g. Experiment 1 and Experiment 2) did not find the effect. Another important point is that, in contrast to the effect for CSs presented for 1000 ms, the effect in the 20 ms presentation time condition was not robust to additional—exploratory—analyses. Including only a few additional participants (i.e. those who knew one or more of the CS stimuli presented for 1000 ms but not those presented for 20 ms) sharply reduced the statistical evidence for an EC effect in the 20 ms condition (i.e. the Bayes factor indicated merely anecdotal evidence). Nevertheless, the EC effects on the evaluation of CSs presented for 20 ms are interesting and give reason to invest additional efforts into the question whether a cross-modal setting to achieve simultaneous presentation of CS and US—as used in this paper—might be well suited for automatic EC effects.

It is additionally worth discussing the order effect of our dependent variables. For CSs presented for 20 ms, the evaluation EC effect was smaller when evaluation was administered after (as compared to before) the choice task. One possible explanation for this pattern of results is a consistency effect in the choice-first group: participants may have evaluated the CSs in line with their previous choices; if, as our results indicate, there was no EC effect on choice, these choices were likely to be random (i.e. equally likely to be consistent as inconsistent with US valence), and this may have masked, or interfered with, the small EC effect on evaluations. This is in line with research showing that choices can influence the preference of initially equally valued items [62] (but note that this finding has recently been critically discussed, and possible boundary conditions were suggested for the influence of choice on preferences [63]).

## 5. General discussion

We set out to test the hypothesis that simultaneous CS-US presentation might be beneficial for EC with briefly presented CSs. To achieve a simultaneous CS-US presentation that guaranteed that the visual attention could be directed towards the briefly presented CS, we implemented a cross-modal paradigm with auditive USs and visual CSs. In the first two studies, we did not observe an EC effect for briefly presented CSs: If anything, we observed some evidence for the *absence* of an EC effect for briefly presented CSs. However, interference arising from the CS identification task—which was prompted after every trial in the learning phases of Experiments 1 and 2—as well as lack of statistical power, could be reasons for not finding an EC effect in these conditions. We therefore omitted the CS identification task in a high-powered Experiment 3, and we additionally introduced a slightly delayed CS onset to create a more simultaneous experience of CS and US. In this third study, we found some evidence for an EC effect for briefly presented CSs, which appeared to be contingent on our—registered—exclusion criteria.

One important limitation is that CSs were not presented truly subliminally in any of the presented studies. CS identification was above chance for all the realized conditions, such that the observed EC effects might have been driven by some participants' conscious perception of some of the CS presentations. Awareness of the contingency between these CSs and the auditive USs may therefore have formed consciously and might underlie the EC effects found in the brief presentation condition. It therefore does not follow that the process underlying the EC effect with briefly presented CSs in Experiment 3 must have been an automatic one. The notion that only a few stimuli were consciously perceived by perhaps only a small subset of participants could also explain the small effect size found for CSs presented for 20 ms in Experiment 3, which could arise from a mixture of an average-sized EC effect contributed by participants who saw the briefly presented stimuli and a null EC effect of participants who did not see the stimuli. Nevertheless, the results of Experiment 3 give reason to further investigate the possibility of an EC effect with truly subliminal CS presentation.

The core proposition of this paper was that a simultaneous presentation of CS and US might be beneficial for EC effects with briefly presented CSs. Through a cross-modal EC procedure with visually presented CSs and auditive USs, we attempted to achieve a simultaneous experience of CS and US. Using this procedure an EC effect with briefly presented—but above-chance visible—CSs was obtained, which is in contrast to the findings by Stahl and colleagues [24]. However, the present study does not address whether the procedural changes indeed had the discussed causal effects, due to the non-experimental manipulation of the (presence versus absence of the) CS identification task, and due to the lack of evidence regarding the effect (or its absence) of slightly delaying the CS onset on EC with briefly presented CSs. Future research should more directly address the possibility that a simultaneous experience of CS and US might be beneficial for EC effects to occur [26], and that a CS identification task to assess visibility, administered after every trial, might interfere with EC for briefly presented CSs. Perhaps most importantly, the present work found a small EC effect with briefly presented CSs, suggesting that the search for a set of enabling conditions for subliminal EC effects might prove worthwhile, and that the cross-modal paradigm proposed here might be a useful method for future studies.

## Figures and Tables

**Figure 8.**
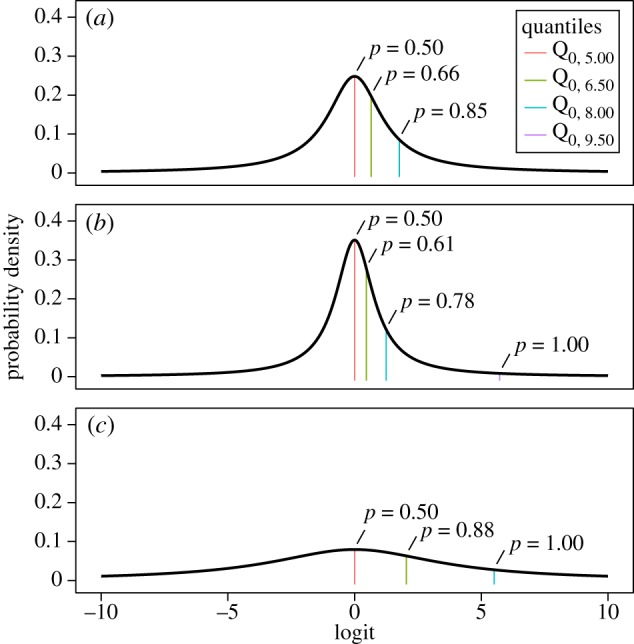
Priors for the Bayesian logistic mixed effects regression models of two-alternative forced choice responses. Coloured lines represent distribution quantiles; annotated probabilities represent the resulting probability of choosing a positively paired CS starting from chance level (*p* = 0.5).

## Footnotes

1 The experiment was programmed using PsychoPy [33].

2 We, furthermore, used the R packages *afex* [44], *BayesFactor* [45], *brms* [41], *fitdistrplus* [46], *ggplot2* [47], *lme4* [48], *lsmeans* [49], *papaja* [50] and *rstan* [51,52].

3 One of the CSs was highly visible even with brief presentations. In an attempt to reduce its visibility, we modified the image file to reduce the contrast of that stimulus. A small pilot study (*N *= 7), where we presented all eight CSs for 20 ms 10 times and asked participants to identify the CS from a list of all eight CSs, showed that the visibility of this stimulus was substantially reduced but still elevated compared to the other CSs (mean visibility = 0.66).

## Data accessibility

All data files, experimental scripts, and analysis scripts can be found at https://osf.io/cx5eh/.

## Appendix A

Sets of IADS sounds used in Experiment 1: Valence Positive, Neutral, Negative (table 2).

**Table 2.** Sound-Nr [32].

positive	neutral	negative
110	109	278
172	171	279
725	206	285
809	221	296
810	270	501
811	365	624
815	367	625
816	368	711
817	375	712
820	722	719

## Appendix B

Priors for the Bayesian logistic mixed effects regression models of two-alternative forced choice responses (figure 8).

## Appendix C

Mean CS visibility (Experiment 2 and Experiment 3).

Mean Visibility scores of each CS in Experiment 2 (chance level = 0.250, *N* = 37) and pilot of Experiment 3 (chance level = 0.125, *N* = 7) and the presentation time for each stimulus as used in Experiment 3 (table 3).

**Table 3.** Mean CS visibility.

CS	visibility study 2	visibility pilot	set (ms)
03.png	0.512	0.400	1000
08.png	0.540	0.329	1000
14.png	0.900	0.657	1000
22.png	0.475	0.400	1000
04.png	0.438	0.200	20
20.png	0.400	0.271	20
50.png	0.356	0.129	20
51.png	0.423	0.243	20
